# Effects of Hydrogen Peroxide Produced by Catechins on the Aroma of Tea Beverages

**DOI:** 10.3390/foods11091273

**Published:** 2022-04-27

**Authors:** Jie-Qiong Wang, Ying Gao, Dan Long, Jun-Feng Yin, Liang Zeng, Yan-Qun Xu, Yong-Quan Xu

**Affiliations:** 1 Tea Research Institute Chinese Academy of Agricultural Sciences, Key Laboratory of Biology, Genetics and Breeding of Special Economic Animals and Plants, Ministry of Agriculture and Rural Affairs, 9 South Meiling Road, Hangzhou 310008, China; wangjieqiong@tricaas.com (J.-Q.W.); yinggao@tricaas.com (Y.G.); yinjf@tricaas.com (J.-F.Y.); 2 College of Food Science, Southwest University, Chongqing 400715, China; 3 Food Research Institute, Hongsheng Beverage Group, Hangzhou 311200, China; dan.long@h-shgroup.com; 4 College of Biosystems Engineering and Food Science, Ningbo Research Institute, Zhejiang University, Ningbo 315100, China; xuyanqun@zju.edu.cn

**Keywords:** green tea beverages, EGCG, hydrogen peroxide, linalool, flavor

## Abstract

Hydrogen peroxide has a significant effect on the flavor of tea beverages. In this study, the yield of hydrogen peroxide in (–)-epigallocatechin gallate (EGCG) solution was first investigated and found to be significantly enhanced under specific conditions, and the above phenomenon was amplified by the addition of linalool. Then, an aqueous hydrogen peroxide solution was added to a linalool solution and it was found that the concentration of linalool was significantly reduced in the above-reconstituted system. These findings were verified by extending the study system to the whole green tea infusions. The results suggested that the production of hydrogen peroxide in tea beverages may be dominated by catechins, with multiple factors acting synergistically, thereby leading to aroma deterioration and affecting the quality of tea beverages. The above results provided a feasible explanation for the deterioration of flavor quality of green tea beverages with shelf life.

## 1. Introduction

Recently, tea drinks have been increasingly sought after by the public for their excellent health benefits [1,2] and favorable flavor. In particular, catechins, which are abundant in green tea beverages, are the most biologically active substances in tea beverages and are crucial players in the formation of the superior and unique flavor of tea beverages [3]. In addition, the aroma of tea beverages is also a key factor in attracting consumers. Although volatiles only account for a notably small fraction, they play an extremely important role in the sensory attributes and quality formation of tea beverages [4].

Nonetheless, the long-standing problem of poor flavor stability inherent in pure tea beverages remains a concern, with green tea beverages being the most affected. During the autoclave process and the long-term shelf life of green tea beverages, a series of chemical reactions occur, such as pigment degradation, oxidized polymerization of catechins, and oxidative degradation of amino acids, resulting in significant changes in flavor (including taste and aroma) and color of tea beverages [5]. Previous studies have found that the types of water [6], water quality [7], tea variety [8], fresh leaf tenderness [9], tea processing [10], and polyphenol concentration [5] all affect the sensory quality and flavor stability of green tea beverages. However, a point worth noting is that the stability of flavor quality of green tea beverages is more or less related to the hydrogen peroxide produced by the oxidation of catechins. Dou et al. [11] pointed out that catechins and the resulting hydrogen peroxide were the key causes of the changes in the aroma composition of green tea beverages.

Hydrogen peroxide is a reactive oxygen intermediate produced by the stepwise reduction of molecular oxygen. It is also a metabolite produced by aerobic cells and is a key secondary messenger signaling molecule in the regulation of many biochemical reactions [12]. It has been suggested that the antimicrobial activity of honey [13] and coffee [14] are attributed to hydrogen peroxide produced by oxidoreductase enzymes and the Maillard reaction, respectively. Furthermore, several previous studies have also reported the production of hydrogen peroxide from different beverage systems. For example, some polyphenol-rich beverages such as green tea, black tea, cocoa, and wine have been reported to produce hydrogen peroxide and are associated with the redox activity of polyphenols [15,16]. It has also been observed that catechins generate large amounts of hydrogen peroxide under both aerobic heating conditions [17] and in different cell culture systems [18]. Alternately, Mitsugu et al. [2] found that green tea and black tea beverages also produced large amounts of hydrogen peroxide even under mild conditions (pH 7.4, temperature 37 °C).

It was thus assumed that hydrogen peroxide may be produced in tea beverages in the presence of oxygen and any redox substrate (e.g., catechins) and that the yield of hydrogen peroxide was significantly disturbed under the corresponding environmental factors. Coupled with that, most previous studies have focused on the production of hydrogen peroxide by monomers or polyphenols under other physiological conditions, whereas fewer studies have been conducted on pure water systems or how hydrogen peroxide affects the flavor of tea beverages. Linalool (floral, spicy, and woody), as one of the key aroma-contributing components in green tea beverages, exerted a great influence on the flavor formation process of tea beverages [11].

As such, the aim of the research was to investigate the production of hydrogen peroxide and changes in linalool in pure water (hydrogen peroxide), linalool–(–)-epigallocatechin gallate (EGCG) (hydrogen peroxide), and hydrogen peroxide–linalool systems (linalool) of catechins, which were subsequently extended to green tea infusions to further investigate the inter-relationship between catechins, hydrogen peroxide, and key aroma components. These results could provide some explanations for the shelf-life flavor quality deterioration of tea beverages (especially green tea beverages), to better develop the stability control of tea beverages, which has practical significance.

## 2. Materials and Methods

### 2.1. Chemicals

EGCG, (–)-epicatechin (EC), (–)-epigallocatechin (EGC), (–)-epicatechin gallate (ECG), and gallic acid (GA) used in the experiment (≥96%) were purchased from Sigma-Aldrich (Shanghai, China). Ferrous sulfate heptahydrate (FeSO_4_) was purchased from Quzhou Juhua Reagent Co., Ltd. (Zhejiang, China). Copper (II) sulfate (CuSO_4_) was obtained from Shanghai Zhen Xing Reagent Factory (Shanghai, China). Potassium chloride, citric acid monohydrate, and absolute ethanol (AR) were obtained from Shanghai Lingfeng Chemical Reagent Co., Ltd. (Shanghai, China). Disodium hydrogen phosphate dodecahydrate, butylated hydroxyanisole (BHA, 98%), and 2,6-di-*tert*-butyl-4-methylphenol (BHT, ≥99.9% (GC)), and linalool (≥96.0% (GC)) were purchased from Shanghai Aladdin Biochemical Technology Co., Ltd. (Shanghai, China). Vitamin C (VC, AR) was purchased from Guangzhou Jinhuada Chemical Reagent Co., Ltd. (Guangdong, China). *Tert*-butyl hydroquinone (TBHQ, ≥98%), trifluoroacetic acid (AR, 99.0%), and iron (III) chloride (CP) were obtained from Shanghai Macklin Biochemical Co., Ltd. (Shanghai, China). Acetone and 30% hydrogen peroxide were purchased from Sinopharm Chemical Reagent Co., Ltd. (Shanghai, China). Folin and Ciocalteu’s phenol reagent was acquired from Wuhan Baiteng Ruida Biological Technology Co., Ltd. (Wuhan, China). DPPH (2,2-diphenyl-1-picrylhydrazyl), ABTS (2,2′-azino-bis(3-ethylbenzothiazoline-6-sulfonic acid)), Trolox (6-hydroxy-2,5,7,8-tetramethylcoromane-2-carboxylic acid), and potassium persulfate were obtained from Sigma-Aldrich (St. Louis, MO, USA). 2,4,6-Tripyridyl-*s*-triazine (BR, 98%) was bought from Shanghai Yuanye Biological Technology Co., Ltd. (Shanghai, China). Methanol (MeOH, HPLC grade), acetonitrile (ACN, HPLC grade), and acetic acid (AA, HPLC, ≥99.9%) were purchased from Merck (Darmstadt, Germany). *n*-alkanes (C7–C40) and ethyl caprate (99%) were from Beijing Zhongsheng Ruitai Technology Co., Ltd. (Beijing, China). A hydrogen peroxide detection kit was purchased from Shanghai Biyuntian Biotechnology Co., Ltd. (Shanghai, China). Pure water used in the experiment was from Hangzhou Wahaha Group Co., Ltd. (Hangzhou, China).

### 2.2. Preparation of Tea Samples

#### 2.2.1. Preparation of the Monomer Reaction System

The samples were mainly prepared from three parts, namely, the catechin reaction system, linalool–EGCG reaction system, and hydrogen peroxide–linalool reaction system. Among them, the first part was mainly carried out from seven aspects, namely, (1) catechin species, (2) catechin concentration, (3) heat treatment time, (4) heat treatment temperature, (5) pH, (6) metal ions, and (7) antioxidants. The second part was mainly carried out considering (8) water bath times and (9) metal ions, whereas the last part tested the (10) hydrogen peroxide–linalool mixture ratio, (11) linalool concentration, (12) water bath time, (13) water bath temperature, and (14) metal ions. For details, refer to Table S1.

#### 2.2.2. Preparation of the Tea Infusion Reaction System

The extraction method of Longjing green tea (one bud and two leaves, Hangzhou, Zhejiang) was slightly modified from Fu et al. [19]. For experimental purposes, the tea polyphenol concentration of the original tea infusions obtained in the modified method was eventually diluted to 250, 500, 750, and 1000 mg/L, then the diluted tea infusions was placed in a water bath at 95 (±2) °C for 40 min to simulate the storage process of tea beverages, after which it was quickly removed, and the hydrogen peroxide yield and antioxidant capacity of the samples were measured after cooling in an ice water bath. The preparation method and tea infusion extraction method of the test raw material used in the validation test, i.e., baked green tea, can be referred to in Wang et al. [20] and Fu et al. [19], respectively, after which the key catechin and aroma components were measured.

### 2.3. Determination of Hydrogen Peroxide

The hydrogen peroxide in the samples was determined using the hydrogen peroxide detection kit, with reference to Gao et al. [21]. The hydrogen peroxide detection reagent was first melted on ice or in an ice water bath, then 50 μL of sample or standard was placed in the test wells or tubes, followed by 100 μL of hydrogen peroxide detection reagent in each well, gently shaken or tapped to mix, and left at room temperature (15–30 °C) for 30 min. The concentration of hydrogen peroxide in the sample was calculated from a standard curve.

### 2.4. Determination of Aroma Compounds

The method for the detection of aroma components can be referenced in the literature [20]. A 0.5 g tea sample was sealed in a 20 mL glass vial and ethyl caprate (internal standard, 10 μL, 10 mg/L, not added for the monomer test of Section 2.2.1), and boiling deionized water (5 mL) was added sequentially. After the mixture was equilibrated for 5 min, the vial was transferred to a water bath at 60 °C and the headspace volatiles were absorbed by divinylbenzene/carboxen/polydimethylsiloxane (50/30 μm DVB/CAR/PDMS, Stable flex (2 cm)) coating fiber (Supelco, Inc., Bellefonte, PA, USA) for 60 min. Later, the volatiles were desorbed in a gas chromatography–mass spectrometry (GC–MS) injector at 250 °C for 5 min.

An Agilent 6890 gas chromatograph with an Agilent HP 5975 MSD ion trap mass spectrometer (Wilmington, DE, USA) was used along with a DB-5MS capillary column (30 m × 250 μm × 0.25 μm) for the analysis of volatiles in tea infusions. The gas chromatography conditions were as follows: inlet temperature of 250 °C; speed of 1.0 mL/min; and splitting ratio of 15:1, with high purity helium (99.999%) used as the carrier gas. The temperature program was: hold at 40 °C for 2 min, ramp up to 85 °C at 2 °C/min and hold for 2 min, ramp up to 180 °C at 2.5 °C/min and hold for 2 min, and ramp up to 230 °C at 10 °C/min and hold for 2 min. The ion source temperature was 230 °C and mass spectrometry was performed in EI mode at 70 eV with a mass scan range of 40–400 *m*/*z*. It is worth mentioning that the determination of linalool concentration in the Section 2.2.1 monomer test was calculated by establishing a standard curve.

### 2.5. Determination of Catechin Content

Based on Fu et al. [19], the content of catechins in the samples was determined. HPLC (Shimadzu LC-2010A HPLC system, Shimadzu Corporation, Kyoto, Japan) was used to analyze the catechin content in tea infusion samples. Before being injected, the tea infusions were filtered through a 0.45 μm Millipore filter. The injection volume was 10 μL. The detection wavelength of the samples was 280 nm. Meanwhile, a Symmetry C18 column (5 μm, 4.6 mm × 250 mm) was used for the chromatographic separation. The column temperature was set at 40 °C. Mobile phase A was H_2_O with 2% AA and mobile phase B was ACN. The elution gradient started at 6.5% for phase B (B) and increased linearly to 15% B at 16 min, 25% B at 25 min, held for 0.5 min, then decreased to 6.5% B at 30 min, followed by equilibration with 6.5% B for 5 min. The total run time was 35 min and the flow rate was 1 mL/min. The concentration of catechins in the sample was calculated by establishing a concentration–peak area standard curve.

### 2.6. Antioxidant Activity Assay

The antioxidant capacity of the samples was determined using the DPPH and ABTS free radical scavenging ability with Trolox equivalent antioxidant capacity. The total antioxidant capacity was characterized by mg Trolox equiv/mL. For both assays, methanol was used as the negative control. The reaction mechanism of the former method was mainly based on the reduction in the free radical DPPH, and the reaction mechanism of the latter method was the reduction in the radical cation of the ABTS that easily decolorized the solution. The specific detection method was revised to a minor extent based on Sun et al. [22].

### 2.7. Statistical Analysis

All experiments were performed at least in triplicate. IBM SPSS Statistics (version 25.0, IBM Corp., Armonk, NY, USA) was employed for analysis of variance, and Origin (version 2018, Origin Lab Corp., Northampton, MA, USA) was used for graphing.

## 3. Results and Discussion

### 3.1. Analysis of Hydrogen Peroxide Formation from Catechins under Various Reaction Conditions

**Figure 1:**
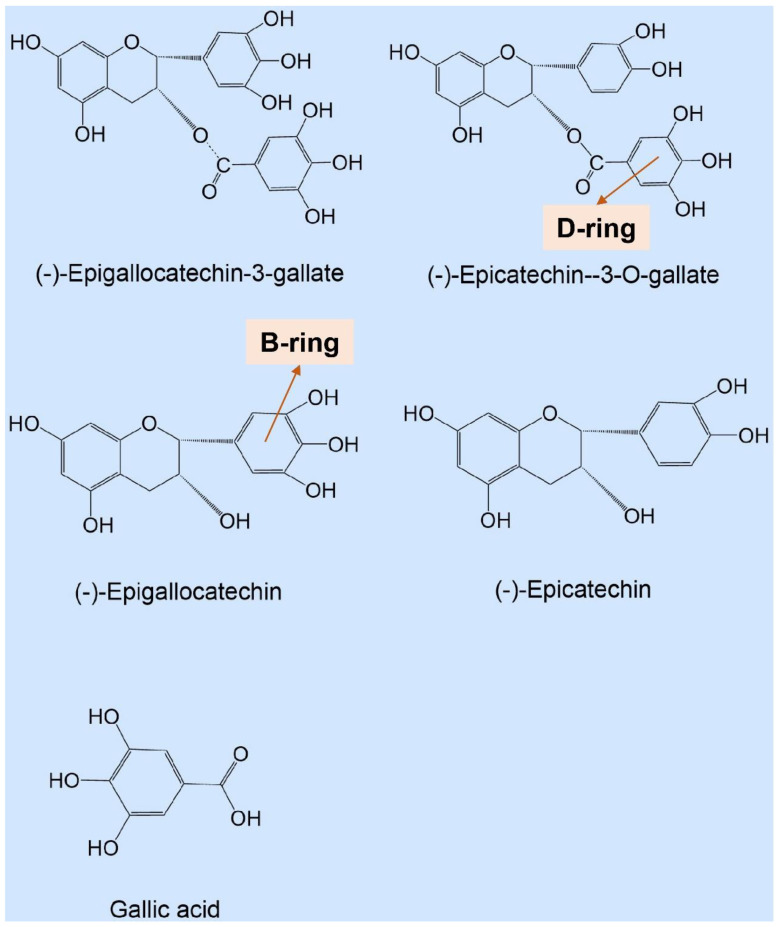
The structure of gallic acid and several catechins discussed in the paper.

During the processing and shelf-life storage of tea beverages, hydrogen peroxide can be derived from the natural oxidation of catechins. Simultaneously, hydrogen peroxide, as a strong oxidizing agent, can expedite the oxidation of catechin compounds, forming a vicious circle of flavor quality [23]. As such, to characterize the production of hydrogen peroxide by catechins, the hydrogen peroxide production capacities of several epi-catechins (EC, ECG, EGC, and EGCG) and GA are shown in Table 1, and the structures of several catechins discussed in the paper are presented in Figure 1. Each catechin sample (100 μM) was incubated in pure water for 2 h at 50 °C, and the production of hydrogen peroxide was subsequently determined. The results showed that the presence of hydrogen peroxide was detected in the solutions of different catechins, and the yield was EGCG > EGC > ECG > EC > GA. Nakayama et al. [24] also reported that the hydrogen peroxide yield of catechins in the 25 mM phosphate buffer (pH = 7.0) system after 60 min of water bath at 37 °C was: EGCG (191 μM) > EGC (179 μM) > GA (28 μM) > EC (21 μM). According to our results, EGCG generated the largest amount of hydrogen peroxide, 127.000 μM, followed by EGC (51.500 μM), whereas GA produced the least amount of hydrogen peroxide, 1.417 μM, reaching only 1.12% of the hydrogen peroxide yield of EGCG (Table 1). Thus, it could be deduced that the yield of hydrogen peroxide was related to the structure of the catechins (Figure 1). Catechins with the gallyl moiety in the catechin B-ring tended to produce more hydrogen peroxide than catechins with the galloyl moiety in the D-ring under the same conditions. The gallyl moiety had a greater effect on the hydrogen peroxide production than the catechol moiety, and the galloyl moiety had a greater effect than the hydroxyl group.

**Figure 2:**
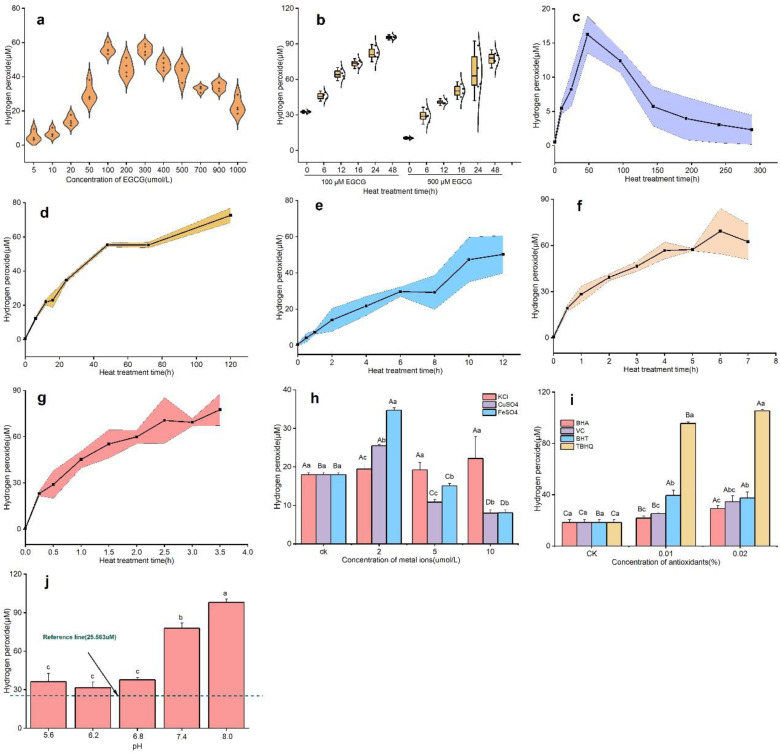
(**a**) Violin diagram of the dose dependency of production of hydrogen peroxide by EGCG (The four repetitive assay data were presented as scattered points in a violin plot pattern). (**b**) Time-course box plot of hydrogen peroxide production at different concentrations of EGCG. (**c**–**g**) Changes in hydrogen peroxide production by EGCG under different temperature gradients of 25 °C (**c**), 37 °C (**d**), 50 °C (**e**), 70 °C (**f**), and 90 °C (**g**) treatments. (**h**–**j**) Effects of metal ions (**h**), antioxidants (**i**), and pH (**j**) (with the numbers 1–5 indicating pH 5.6, 6.2, 6.8, 7.4, and 8.0, respectively, and the reference line indicating that the solvent was pure water) on hydrogen peroxide production by EGCG. Different lowercase letters on the bar graphs indicate significant differences within groups, and different uppercase letters indicate significant differences between groups.

As can be seen from Figure 2a, the amount of hydrogen peroxide production did not always show a linear increase with increasing EGCG concentration (from 0 to 1000 μM) but showed an overall “double hump” pattern (one point was 56.131 μM (100 μM) and another point was 56.012 μM (300 μM)). A highly significant positive correlation (*r* = 0.955^**^, *p* < 0.01) between EGCG concentration and hydrogen peroxide yield was observed below the first point (100 μM), whereas a highly significant negative correlation (*r* = –0.915^**^, *p* < 0.01) was observed above the second point (300 μM). This phenomenon might be explained by the fact that the concentration of hydrogen peroxide produced by the system reached an equivalent concentration at a specific EGCG concentration, i.e., an equilibrium was reached between the production and decomposition of hydrogen peroxide [2,25]. The amount of hydrogen peroxide produced by EGCG increased gradually with the extension of the water bath process (from 0 to 48 h), and the yield of hydrogen peroxide was lower at 500 μM than at 100 μM EGCG concentration at a given time (Figure 2b). However, the yield of hydrogen peroxide changed more rapidly in the former concentration condition than in the control treatment (0 h), reaching 86.45%, whereas the latter only increased by 66.00% compared with the control treatment (0 h), which further validated the phenomenon in Figure 2a.

The variation of hydrogen peroxide yield at the five selected temperature points (25, 37, 50, 70, and 90 °C) is shown in Figure 2c–g. It was found that the trend of hydrogen peroxide yield at 25 °C (showing a parabolic trend) was significantly different from the other four temperatures (showing a linear increase). At 25 °C, the hydrogen peroxide yield was extremely close to 0 at the beginning and reached the highest point (13.095 μM) after 48 h in a water bath; after that, the yield of hydrogen peroxide decreased gradually with the extension of the water bath time until it was close to 0 (2.330 μM) (Figure 2c). The result was comparable to the trends of hydrogen peroxide in green tea beverages (with added antioxidant combination) [11] and sugar-free oolong tea beverages [26] during storage in the tea beverage system. The reason may be that the hydrogen peroxide yield reached the maximum at 48 h incubation; after that, with the extension of incubation time, the O^2–^ or hydrogen peroxide produced in the system was gradually removed by EGCG or other degradation products, so that it eventually approached zero.

In contrast, at other temperatures (37, 50, 70, and 90 °C), the yield of hydrogen peroxide in the system gradually increased with increasing water bath time (Figure 2d–g), presumably related to the autoxidation of EGCG in the system. To test the above conjecture further, the contents of the major catechins produced in the EGCG reaction solution were assayed as in Table S2. It can be observed from the table that the EGCG in the system solution with heat treatment was autoxidized to GCG, ECG, and GA. The trend of ECG generated from different concentrations of EGCG (100 and 500 μM) was different with the increase in the water bath time, although the content of GCG and GA exhibited an increasing trend at both concentrations. The trends of ECG generated by different concentrations of EGCG autoxidation during the heat treatment are presented in Figure S1. It was found that the concentration of EGCG had a large effect on the autoxidation of the system product ECG, which presented different trend lines. Degree of autoxidation of EGCG of EGCG in the 100 μM EGCG system reached two (70 °C) to three (90 °C) times the initial concentration, and the autoxidation was more complete compared with the high concentration (100 μM) of the EGCG system. Despite the transient accumulation of ECG during the water bath phase, EGCG degradation still dominated, resulting in a greater rate of hydrogen peroxide production than decomposition, contributing to the progressive accumulation of hydrogen peroxide at temperatures of 70 and 90 °C.

From Figure 2h, it was observed that the addition of KCl promoted the yield of hydrogen peroxide compared with the control (CK) (without the addition of metal ions), but it did not reach a significant level between the different concentrations. However, after the addition of CuSO_4_ and FeSO_4_, a significant increase in hydrogen peroxide concentration was found. At a concentration of 2 μM, the yield of hydrogen peroxide increased significantly (the rate of change of FeSO_4_ was greater than for CK, reaching 48.20%), whereas when the concentrations of 5 and 10 μM were chosen, the yield of hydrogen peroxide decreased instead and also reached a significant level (*p* < 0.05). The results were somewhat similar to those of Grzesik et al. [25], which indicated that the addition of lower concentrations of ascorbic acid (less than 0.5 mM) to tea brewed in tap water significantly increased the yield of hydrogen peroxide in the system, but at higher concentrations (1 mM) it decreased its yield instead, which was not the case in deionized water. This result may be related to the occurrence of transition metal ions in the water. It was no coincidence that the aroma of tea infusions deteriorated at the concentration of Fe^2+^ of only 0.1 ppm, indicating that the aroma of tea infusions was extremely sensitive to Fe^2+^ [27]. The appearance of Fe^2+^ was dose-related and aggravated the deterioration of heat-induced aromas [21]. This indicated that the autoxidation of EGCG in the system was probably also driven by the type and concentration of metal ions.

Moreover, it was found that the addition of the four selected antioxidants increased the yield of hydrogen peroxide, and the yield of hydrogen peroxide was: TBHQ > BHT > VC > BHA (Figure 2i). The production of hydrogen peroxide increased gradually with the increase in antioxidant concentration, with the highest being TBHQ, which reached 105.375 μM, an increase of 82.68% compared with the CK (no antioxidant added). It was assumed that the above antioxidants may not effectively inhibit the autoxidation of tea polyphenols, or the oxidation of the antioxidants themselves may lead to enhanced production of hydrogen peroxide. Podmore [28] found that L Vitamin C only exerted a certain antioxidant effect during the first month of beverage storage, after which oxidation accelerated. Ascorbic acid could produce hydrogen peroxide in the presence of molecular oxygen, but if ascorbic acid could mediate a net increase in reactive oxygen species, it could also act as a pro-oxidant [29]. In addition, the effect of different pH gradients on the yield of hydrogen peroxide was investigated by changing the reaction medium of EGCG, as shown in Figure 2j. It was found that the yield of hydrogen peroxide increased significantly with increasing pH (5.6–8.0), especially when the pH ≥ 7.4 reached a significant level (*p* < 0.05) compared with the previous pH (< 7.4). It was assumed that the production of hydrogen peroxide by catechins in the intestine may be high due to their intense autoxidation in an alkaline environment.

These results initially explored the yield of hydrogen peroxide in aqueous catechin systems under different treatment conditions and showed that specific treatment conditions significantly enhanced the yield of hydrogen peroxide. However, the effect of aroma, another important substance in tea beverages that regulates quality, on the yield of hydrogen peroxide production from key catechins was not yet known.

### 3.2. Effect of Key Aroma Component Linalool on Hydrogen Peroxide Production by EGCG

In the above study, it was found that the amount of hydrogen peroxide produced by EGCG varied greatly in different environments. In a previous study, Dou et al. [11] found that hydrogen peroxide was also one of the key factors affecting the aroma variation of tea beverages by establishing multiple regression equations between key taste substances such as total phenols, catechins, hydrogen peroxide, free amino acids, and caffeine, and key aroma substances such as linalool, geraniol, β-ionone, linalool oxide, hexanal, benzaldehyde, and nerolidol, which reached a significant level. Therefore, the next study focused on the key aroma component, linalool, to investigate its effect on hydrogen peroxide production by EGCG.

**Figure 3:**
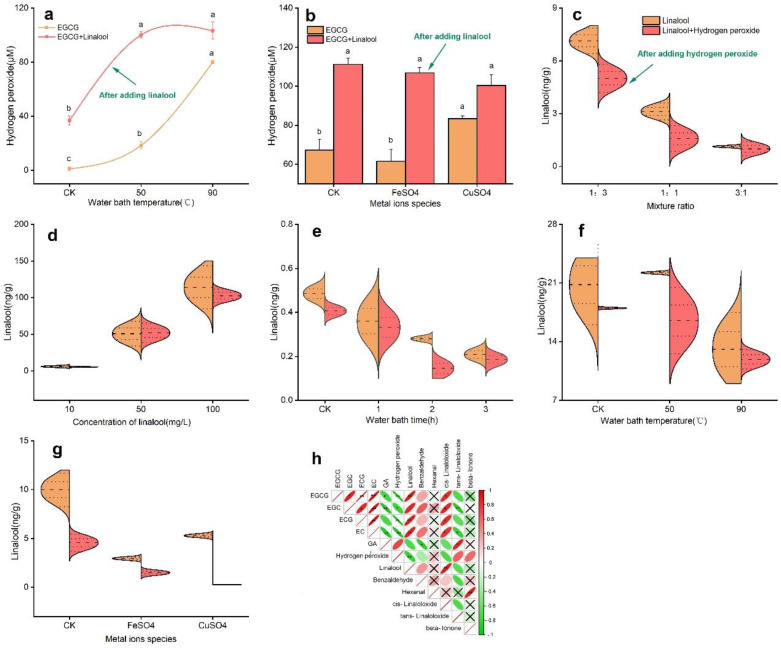
(**a**,**b**) Amount of hydrogen peroxide produced by the EGCG−linalool solution under water bath conditions (**a**) and metal ions (**b**). (**c**–**g**) Variation of linalool concentration in linalool−hydrogen peroxide reaction solution under different mixing ratios (**c**), linalool concentration (**d**), water bath time (**e**), water bath temperature (**f**), and metal ions (**g**). (**h**) Heat map of the correlation between the yield of hydrogen peroxide in baked green tea and key catechins and aroma components (* indicates significance at the 0.05 level in Pearson correlation analysis, and ** indicates significance at the 0.01 level in Pearson correlation analysis). Different lowercase letters on the bar graphs indicated significant differences within groups.

From Figure 3a, it can be seen that before the addition of linalool, the amount of hydrogen peroxide produced by EGCG showed a linear increase with the increase in temperature; nevertheless, this change was more significant with the addition of linalool than with the control group (*p* (two-tailed) < 0.05). The rate of change of hydrogen peroxide produced before and after the addition of linalool reached 97.05% (control), 81.60% (50 °C), and 22.52% (90 °C). Apparently, the amount of hydrogen peroxide produced by the linalool–EGCG reaction system was more correlated with the temperature of the process.

The next study found that the addition of metal ions to EGCG could achieve the same effect as the water bath treatment, as shown in Figure 3b. Before the addition of linalool, the amount of hydrogen peroxide produced by EGCG was reduced compared with CK (without the addition of metal ions); however, with the addition of linalool, the yield of hydrogen peroxide increased significantly, reaching more than 100 μM, but the difference in the yields under different metal ion conditions was not significant.

The above results showed that the linalool–EGCG solution treatment (heating and metal ions, for example) enhanced the generation of hydrogen peroxide. It was thus assumed that the generation of hydrogen peroxide in the tea beverage system was not the result of the oxidative degradation of one substance such as catechin alone, but was most likely the result of the combined action of catechin and other flavor-contributing components so that the generated hydrogen peroxide further reacted with the tea beverage to forge a new flavor. Then, hydrogen peroxide was added to the linalool solution to form a hydrogen peroxide–linalool solution system to explore the effect of hydrogen peroxide on the linalool in the system.

### 3.3. Effect of Hydrogen Peroxide on the Key Aroma Component Linalool

Not only did linalool have a direct effect on the amount of hydrogen peroxide produced by the EGCG solution (discussed above) but the linalool in the system itself also changed. Accordingly, the changes in the concentration of linalool in the hydrogen peroxide–linalool system were further investigated.

In Figure 3c, it is shown that the addition of different volumes of hydrogen peroxide to linalool significantly reduced the concentration of linalool and the rates of change at different ratios were −29.80% (1:3), −50.00% (1:1), and 11.57% (3:1). Therefore, a 1:1 ratio was used as the study subject for subsequent experiments. Subsequently, different concentrations of linalool (10, 50, and 100 mg/L, Figure 3d), bath time (1, 2, and 3 h, Figure 3e), bath temperature (50 and 90 °C, Figure 3f), and addition of metal ions (FeSO_4_ and CuSO_4_, Figure 3g) brought a similar conclusion as above, i.e., the addition of hydrogen peroxide also reduced the concentration of the key aroma substances such as linalool. The maximum rate of change of linalool was found at a linalool concentration of 100 mg/L, a water bath time of 2 h, a water bath temperature of 50 °C, and with the metal ion of Cu^2+^, which were −9.97%, −47.98%, −25.71%, and 94.98%, respectively.

These results indicated that the hydrogen peroxide–linalool system under different environmental conditions significantly reduced the content of linalool. It could be concluded that many material components in the whole tea beverage system, led by hydrogen peroxide, acted together on the aroma components of the system, thus causing aroma deterioration and affecting the tea beverage quality. Because hydrogen peroxide changed significantly during the storage of tea beverages, it could be considered a weather vane for flavor deterioration of tea beverages. In the following study, the above results were verified by extending the study system from a simple monomer system to a complex green tea beverage system.

### 3.4. Analysis of the Formation of Hydrogen Peroxide in Tea Infusions

In the above discussion, it was found that the hydrogen peroxide generated from catechins under different conditions served the whole system (addition of linalool), resulting in significant changes in both hydrogen peroxide and the main aroma component, i.e., linalool. Next, the study medium was extended to the tea infusion system, and green tea infusions were used as the main object in this study to further analyze the generation of hydrogen peroxide in the tea infusion system. It was clear that with the increase in polyphenol concentration, the yield of hydrogen peroxide decreased significantly, whereas the antioxidant capacity increased significantly (Table 2). Moreover, the trends of the results measured using both DPPH and ABTS were consistent, further validating that the phenolics with strong antioxidant capacity in the tea infusions had a good scavenging effect on the hydrogen peroxide produced by the system, which was in agreement with the results of previous studies related to the scavenging ability of phenolics in apple peel on hydrogen peroxide [30].

To further verify the above results, the key catechins, hydrogen peroxide yields, and several key aroma substances in different baking green teas were examined one after another. The variation of EGCG concentration and hydrogen peroxide yield of the key catechins in baked green tea are shown in Table S3. In addition, Figure S2 shows the HPLC chromatograms of the corresponding catechin fractions in the samples. It was observed that the EGCG degraded with the deepening of baking, whereas the yield of hydrogen peroxide increased, and the steamed green tea changed more than the pan-fried green tea. Therefore, a significant negative correlation was also identified between the concentration of EGCG and the yield of hydrogen peroxide, further validating the above results. The processing method of tea leaves (steamed green and fried green) also had a great effect on the production of hydrogen peroxide in the green tea infusion system.

Notably, correlations between the yield of hydrogen peroxide in baked green tea and key catechins such as EGCG, EGC, ECG, EC, and GA, and aroma components such as linalool, benzaldehyde, hexanal, *cis*-linaloloxide, *trans*-linaloloxide, and beta-ionone were also analyzed to further illustrate how hydrogen peroxide in tea beverages affected their flavor quality (mainly taste and aroma quality) in Figure 3h. It can be observed that the yield of hydrogen peroxide in baked tea correlated with key aroma components such as linalool (*p* < 0.05, *r* = −0.808) and key catechins such as EGCG (*p* < 0.01, *r* = −0.975), EGC (*p* < 0.05, *r* = −0.788), ECG (*p* < 0.01, *r* = −0.978), and EC (*p* < 0.01, *r* = −0.935), all reaching significant negative correlations with each other. As such, it could be concluded that the deterioration of the flavor quality of green tea infusions was inextricably linked to the hydrogen peroxide produced mainly by catechins in the system.

### 3.5. Mechanism of Hydrogen Peroxide Production in Tea Beverages

Tea beverages can result in significant hydrogen peroxide production during the shelf life of tea beverages, even when exposed to light alone. For example, hydrogen peroxide was determined as soon as the bottles of tea and coffee were opened [15]. With prolonged incubation, hot- and cold-water extracts of tea, coffee, and cocoa produce hydrogen peroxide greater than 100 μM [15,31,32], at which concentration irreversible oxidative damage to proteins, tissues, and important biomolecules was expected and affected cellular pathways [33]. Although the present experiment also involved hydrogen peroxide yields greater than 100 μM, the yield of hydrogen peroxide in beverages may have been overestimated in the relevant literature because of the possibility of false positive results due to nonspecific reactions during the assay. Therefore, a further evaluation of the produced hydrogen peroxide using a different method, namely, a hydrogen peroxide-sensitive colorimetric method, should follow later. Nevertheless, it still cannot be ruled out that hydrogen peroxide is not toxic to humans, not to mention that it may be converted into more toxic hydroxyl radicals during digestion or in the case of iron ion supply, in the body. Therefore, it becomes particularly important to understand the mechanism of hydrogen peroxide generation in foods and beverages.

**Figure 4:**
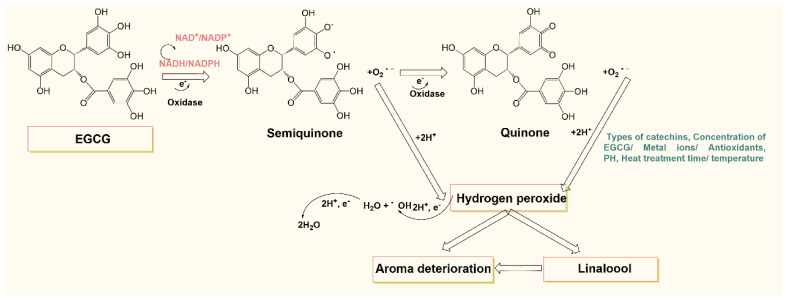
Possible mechanism of aroma quality induced by hydrogen peroxide produced by catechins in tea beverages.

As shown in Figure 4, molecular oxygen was reduced to produce a chemically active intermediate (standard reduction potential is −0.33 V), i.e., a superoxide radical cation. Although the key catechin, EGCG, was oxidized to a semiquinone intermediate, this intermediate NADH/NADPH was converted to NAD^+^/NADP^+^, which incidentally provided one electron. The superoxide radical as an intermediate oxidation state can be reduced to hydrogen peroxide and oxidized to oxygen and has an extremely long half-life, up to several days at 37 °C, which is neither reactive nor toxic and is more easily oxidized by oxygen than the fully reduced catechins [25]. Afterward, the semiquinone and the above-generated superoxide anion O_2_**^•–^** were oxidized again, one electron was involved in the reaction, a quinone was generated, and superoxide was formed again. Subsequently, the superoxide radicals twice underwent a superoxide disproportionation reaction, before and after, with the participation of two hydrogen ions and one electron, leading to the production of hydrogen peroxide.

Hydrogen peroxide has a higher stability than other forms of reactive oxygen species and is probably the most abundant reactive oxygen intermediate [34] in chemical or biological systems. It has been reported that the sulfonic acid group [34], as well as the piperazine ring [35], can reduce molecular oxygen to hydrogen peroxide. Nevertheless, this study found that the gallyl moiety on the EC in tea beverages also played a role in the generation of hydrogen peroxide in the whole tea beverage system. The Fenton reaction then reduces some of the generated hydrogen peroxide either to water and hydroxide ions or directly to water. The majority of the remaining hydrogen peroxide is involved in the formation of flavor substances in tea beverages, albeit with negative effects under certain conditions.

## 4. Conclusions

In this paper, the role of hydrogen peroxide in a tea beverage simulation system was investigated. By investigating the yield of hydrogen peroxide in the EGCG system under different environmental conditions, it was found that specific conditions positively stimulated the yield of hydrogen peroxide, and the addition of the key aroma substance linalool to the above system showed that linalool was a catalyst for the yield of hydrogen peroxide, suggesting that the production of hydrogen peroxide in the simulated tea beverage system may be catechin-dominated with the synergistic effect of multiple factors (environmental factors and aroma substances) as a result.

By adding hydrogen peroxide directly to the linalool solution system, it was found that the linalool concentration was also significantly reduced, indicating that the hydrogen peroxide generated under the stimulation of the abovementioned multiple effects may also affect the aroma substances in the system. Subsequent studies on the tea infusion system further confirmed the good correlation between catechin–hydrogen peroxide–key aroma substances mentioned above. The mechanism of aroma deterioration in tea beverages dominated by hydrogen peroxide in tea beverage systems was also postulated. The results of this study provided a novel idea for the quality control of the shelf life of green tea beverages.

## Figures and Tables

**Table 1:** Amounts of hydrogen peroxide produced during heat-processing of different catechin solutions at 50 °C (different lowercase letters indicate significant differences between mean values the same column (*p* < 0.05)).

Types of Catechins *	Production of Hydrogen Peroxide (Μm)
GA	1.417 ± 0.167 ^d^
EC	6.167 ± 0.694 ^c,d^
ECG	8.417 ± 1.316 ^c^
EGC	51.500 ± 6.501 ^b^
EGCG	127.000 ± 6.633 ^a^

* A total of 4 mL of each catechin solution (100 μM in pure water) was incubated for 2 h at 50 °C. After immediate cooling, the concentration of hydrogen peroxide was quantified as described in Materials and Methods. Data were averages of at least four determinations.

**Table 2:** Analysis of hydrogen peroxide yield and antioxidant activity of Longjing tea at different polyphenol concentrations (different lowercase letters (a, b, c, d) indicate significant differences between mean values the same column (*p* < 0.05)).

Concentration of Tea Polyphenols (mg/L)	Concentration of H_2_O_2_ (μM)	DPPH (M Trolox Equivalents)	ABTS (M Trolox Equivalents)
250	75.111 ± 0.476 ^a^	92.196 ± 3.674 ^b^	60.091 ± 5.233 ^d^
500	58.603 ± 0.825 ^b^	94.801 ± 5.952 ^b^	95.323 ± 6.370 ^c^
750	50.825 ± 2.651 ^c^	101.415 ± 0.000 ^b^	133.290 ± 4.550 ^b^
1000	40.508 ± 0.727 ^d^	112.238 ± 1.701 ^a^	167.234 ± 2.048 ^a^

## Data Availability

The data presented in this study are available within the article.

## Supplementary Materials

The following supporting information can be downloaded at: https://www.mdpi.com/article/10.3390/foods11091273/s1. Table S1: Preparation of tea samples; Table S2: Changes in the content of major catechins produced by the EGCG solution system during heat treatment; Table S3: EGCG content and the amount of hydrogen peroxide produced in green tea with different baking levels; Figure S1: Changes in the content of ECG produced in EGCG system solution during heat treatment. (**A**) Amount of 100 μM of EGCG, heat treatment of 70 °C; (**B**) 500 μM of EGCG, heat treatment of 70 °C; (**C**) 100 μM of EGCG, heat treatment of 90 °C; and (**D**) 500 μM of EGCG, heat treatment of 90 °C; Figure S2: HPLC chromatograms of catechin fraction of green tea with different baking processes.

## Abbreviations

GC–MS	gas chromatography–mass spectrometry
HPLC	High performance liquid chromatography
DPPH	2,2-diphenyl-1-picrylhydrazyl
ABTS	2,2′-azino-bis(3-ethylbenzothiazoline-6-sulfonic acid)
