# Supplementary material for: Effects of Hydrogen Peroxide Produced by Catechins on the Aroma of Tea Beverages
Source: Foods. 2022 Apr 27;11(9):1273. doi: 10.3390/foods11091273 (PMC9102859; doi:10.3390/foods11091273)
Supplement: Supplementary file 1 [file foods-11-01273-s001.zip › foods-1674258-supplementary.pdf]

Supplementary Materials

Effects of Hydrogen Peroxide Produced by Catechins on the Aroma of Tea Beverages

Jieqiong Wang [1, 2], Ying Gao [1], Dan Long [3], Junfeng Yin [1], Liang Zeng [2, *], Yanqun Xu [4], Yongquan Xu [1**]

[1] Tea Research Institute Chinese Academy of Agricultural Sciences, Key Laboratory of Tea Biology and Resources Utilization, Ministry of Agriculture, 9 South Meiling Road, Hangzhou 310008, China.

[2]College of Food Science, Southwest University, Chongqing 400715, China

[3] Food Research Institute, Hongsheng Beverage Group, Zhejiang, 311200, China.

[4] College of Biosystems Engineering and Food Science, Ningbo Research Institute, Zhejiang University, Zhejiang, 315100, China.

Corresponding Authors

[**]Yong-Quan Xu, Tel: +86-571-86650594. Fax: +86 571 86650056. Email: yqx33@126.com.

[*]Liang Zeng, Tel: +86-023-68250374. Fax: +86 023 68251743. Email: zengliangbaby@126.com.

Table S1. Preparation of tea samples.

Different conditions	Sample treatment process
**catechin reaction system**	
(1) Catechins species	EGCG, EC, ECG, EGC and GA monomer powder (purity ≥ 96%) were dissolved in pure water and prepared into a solution with a concentration of 100 μM.
(2) Catechin concentration	Prepare EGCG solutions with concentrations of 5, 10, 20, 50, 100, 200, 300, 400, 500, 700, 900, 1000 μM respectively.
(3) Heat treatment time	the EGCG solutions with concentrations of 100, 500 μM were heated at 50 °C for 0, 6, 12, 16, 24, and 48 h, respectively.
(4) Heat treatment temperature	the EGCG solution with a concentration of 100 μM was heated in a water bath at 25°C for 0, 10, 24, 48, 96, 144, 192, 240, 288 h; at 37°C for 0, 6, 12, 16, 24, 48, 72, 120 h; at 50°C for 0, 0.5, 1, 2, 4, 6, 8, 10, 12 h, respectively; at 70°C for 0, 0.5, 1, 2, 3, 4, 5, 6, 7 h respectively; at 90°C for 0, 0.25, 0.5, 1, 1.5, 2, 2.5, 3, 3.5 h respectively.
(5) pH	the EGCG was dissolved in the prepared disodium hydrogen phosphate-citrate buffer (0.1M citric acid and 0.2M disodium hydrogen phosphate) of pH 5.6, 6.2, 6.8, 7.4 and 8.0 to form a 100 μM buffer solution of EGCG. After that, 4 mL of the prepared solution (1-5) was placed in a 15 mL centrifuge tube, in a water bath at 50 °C (except 4) for 2 h (except 3), followed by immediate removal and cooling to room temperature in an ice water bath, and the hydrogen peroxide yield was to be determined.
(6) metal ions	prepare aqueous solutions of $FeSO_4$, $CuSO_4$, KCl with concentrations of 2, 5, 10 μM, respectively.
(7) Antioxidants	0.01% and 0.02% of antioxidants VC, BHA, BHT, TBHQ were prepared. After that, 6-7 were mixed with aqueous EGCG solution with a concentration of 100 μM in the ratio of 1:10 (i.e., 0.4 mL metal ion solution: 4 mL EGCG solution), 4.4 mL of the mixture was placed in a 15 mL centrifuge tube, 50 °C water bath for 2 h, and then removed for determination.
**linalool-EGCG reaction system**	
(8) water bath time	100 mg/L of linalool was mixed with EGCG at

	the same concentration in the ratio of 1:1 (i.e., 2 mL:2 mL), the mixture was water bath at 50, 90 °C for 1 h (unheated CK was used as control).
(9) metal	100 mg/L of linalool was mixed with EGCG at the same concentration in the ratio of 1:1 (i.e., 2 mL:2 mL), the above mixture was mixed with 5 μM of $FeSO_4$, $CuSO_4$ solution for a second time and water bath at 70 °C for 1 h. Afterwards, the water bath was (8-9) was removed and the ice water bath was cooled to room temperature, and the hydrogen peroxide yield was to be measured.
**hydrogen peroxide-linalool reaction system**	
(10) mixing ratio	A mixture of hydrogen peroxide (100 mM) and linalool (10 mg/L) was prepared in the mixing ratio of 1:3,1:1, 3:1 (i.e., 1 mL: 3 mL, 2 mL: 2 mL, 3 mL: 1 mL) in a water bath at 70°C for 1 h.
(11) linalool concentration	a mixture of hydrogen peroxide (100 mM) and different concentrations ((10, 50, 100 mg/L) of linalool was prepared in the ratio of 1:1 mixture, 70 °C water bath for 1 h.
(12) water bath time	Hydrogen peroxide (100 mM) and linalool (10 mg/L) were mixed in the ratio of 1:1 and bathed in water at 70°C for 1, 2 and 3 h, respectively.
(13) water bath temperature	Hydrogen peroxide (100 mM) was mixed with linalool (10 mg/L) in the ratio of 1:1 and then bathed for 1h at 50°C and 90°C.
(14) metal	5 μM of $FeSO_4$ and $CuSO_4$ were added to a solution of hydrogen peroxide (100 mM) mixed with linalool (10 mg/L) in the ratio of 1:1, and a water bath at 70°C for 1 h. After that, the above samples were (10-14) were removed and cooled to room temperature in an ice-water bath, and the linalool concentrations were to be tested.

Table S2. Changes in the content of major catechins produced by the EGCG solution system during heat treatment (Unit: mg/L, different lowercase letters indicate significant differences between mean values the same column ($p < 0.05$), the same below).

Heat treatment time	100 μM EGCG Solution				500 μM EGCG Solution			
(h)	EGCG	GCG	ECG	GA	EGCG	GCG	ECG	GA
**70 °C**								
0	32.271±2.620^{a}	0.000±0.000^{e}	1.139±0.078^{a}	0.044±0.003^{d}	183.178±2.238^{a}	0.000±0.000^{g}	6.979±0.003^{a}	0.173±0.010^{g}
0.5	28.050±1.279^{b}	0.000±0.000^{e}	1.258±0.002^{a}	0.179±0.045^{cd}	172.471±0.991^{a}	3.268±0.261^{f}	6.964±0.027^{a}	0.322±0.036^{f}
1	26.246±1.164^{b}	1.874±0.277^{d}	1.287±0.022^{a}	0.318±0.066^{c}	169.551±2.508^{b}	5.412±0.439^{e}	6.930±0.010^{a}	0.459±0.045^{e}
2	21.985±1.568^{c}	2.798±0.250^{c}	1.186±0.032^{a}	0.568±0.061^{b}	160.330±1.372^{bc}	7.730±1.389^{d}	6.681±0.076^{b}	0.719±0.085^{d}
3	20.383±2.194^{cd}	3.285±0.197^{b}	1.187±0.016^{a}	0.706±0.101^{b}	156.666±3.070^{c}	9.904±0.506^{c}	6.651±0.013^{b}	0.883±0.050^{c}
4	17.315±3.222^{d}	3.967±0.298^{a}	1.193±0.122^{a}	0.921±0.195^{a}	153.959±2.782^{d}	11.195±0.708^{b}	6.719±0.005^{b}	1.114±0.113^{b}
5	18.238±0.594^{d}	3.880±0.473^{a}	1.189±0.026^{a}	0.923±0.043^{a}	147.813±3.014^{e}	12.818±0.871^{a}	6.632±0.079^{b}	1.304±0.104^{a}
**90 °C**								
0	32.271±2.620^{a}	0.000±0.000^{f}	1.139±0.078^{a}	0.044±0.003^{g}	183.178±2.238^{a}	0.000±0.000^{e}	6.979±0.003^{a}	0.173±0.010^{f}
0.25	25.606±0.718^{b}	3.543±0.335^{e}	1.223±0.001^{a}	0.413±0.054^{f}	165.725±5.224^{b}	8.976±1.167^{d}	6.988±0.217^{a}	0.542±0.101^{e}
0.5	22.571±0.206^{c}	6.013±0.599^{d}	1.147±0.021^{a}	0.654±0.021^{e}	155.431±7.205^{c}	17.281±2.981^{c}	6.837±0.170^{ab}	0.928±0.207^{d}
1	17.687±0.720^{d}	8.531±0.242^{c}	1.042±0.010^{b}	0.978±0.024^{d}	148.862±3.138^{c}	26.278±1.514^{b}	6.555±0.051^{bc}	1.293±0.164^{c}
1.5	15.744±1.108^{de}	9.967±0.796^{b}	0.980±0.012^{bc}	1.132±0.117^{c}	139.535±1.695^{d}	31.284±1.056^{ab}	6.290±0.118^{c}	1.693±0.050^{b}
2	14.192±1.046^{ef}	10.572±1.034^{ab}	0.955±0.040^{cd}	1.336±0.070^{b}	133.693±3.863^{de}	34.142±5.587^{a}	6.371±0.152^{c}	2.062±0.350^{a}
2.5	12.163±0.515^{f}	11.385±1.363^{a}	0.878±0.013^{d}	1.545±0.091^{a}	129.308±1.434^{e}	35.289±6.471^{a}	6.288±0.088^{c}	2.280±0.169^{a}

Table S3. EGCG content and the amount of hydrogen peroxide produced in green tea with different baking levels (SST: steamed tea leaves; SBST: steamed baked spring tea leaves; SAT: steamed autumn tea leaves; SLBAT: steamed and lightly baked autumn tea leaves; SBAT: steamed and baked autumn tea leaves; PST: pan-fired spring tea leaves; PBST: pan-fired and baked spring tea leaves).

Samples	EGCG	Concentration of H_2O_2 (μmol/L)
SST	0.874 ± 0.000^{b}	24.250 ± 0.50^{d}
SBST	0.230 ± 0.000^{g}	48.250 ± 0.250^{a}
SAT	0.707 ± 0.000^{d}	26.125 ± 0.375^{c}
SLBAT	0.491 ± 0.000^{e}	33.125 ± 0.375^{b}
SBAT	0.240 ± 0.000^{f}	48.625 ± 0.375^{a}
PST	1.257 ± 0.001^{a}	2.875 ± 0.125^{f}
PBST	0.829 ± 0.000^{c}	14.375 ± 0.125^{e}

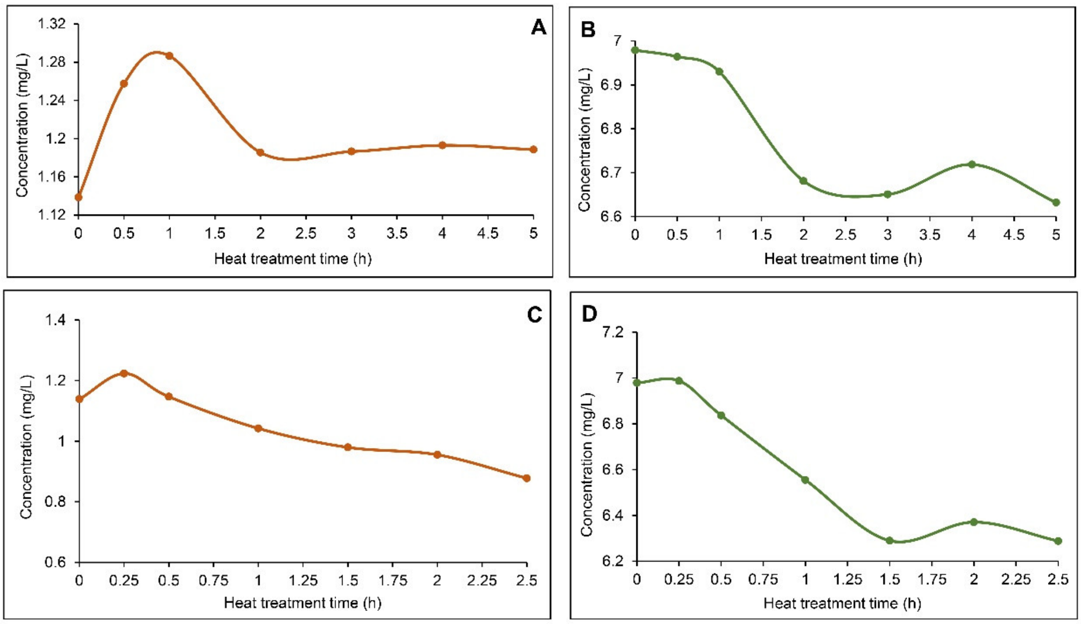

Figure S1. Changes in the content of ECG produced in EGCG system solution during heat treatment. (**A**) 100 μM of EGCG, heat treatment of 70°C; (**B**) 500 μM of EGCG, heat treatment of 70°C; (**C**) 100 μM of EGCG, heat treatment of 90°C; (**D**) 500 μM of EGCG, heat treatment of 90°C. Each point shown in the graph represents the mean of at least three replicates of a single treatment.

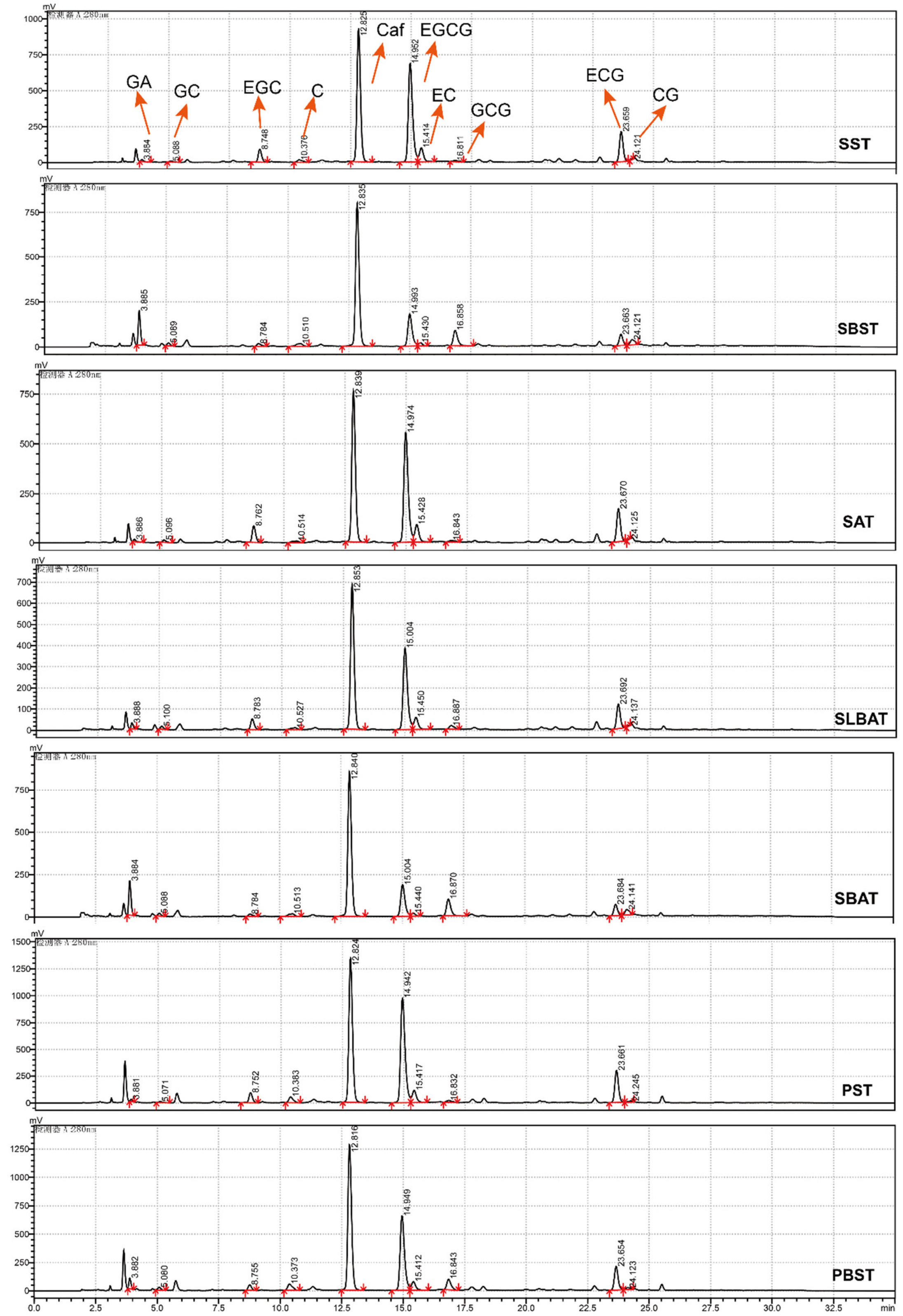

Figure S2. HPLC chromatograms of catechin fraction of green tea with different baking processes.
